# Dynamics of a network of excitatory and inhibitory neurons induced by depolarization block

**DOI:** 10.1186/1471-2202-15-S1-P76

**Published:** 2014-07-21

**Authors:** Christopher Kim, Duane Q Nykamp

**Affiliations:** 1 School of Mathematics, University of Minnesota, Minneapolis, MN, 55455, USA

## 

The balance of excitation and inhibition is important in regulating the network activity. Under pathological conditions the interaction of excitatory and inhibitory neurons can become unbalanced, leading to seizure-like activity in the network. The interplay between excitatory and inhibitory neurons has been studied in rat hippocampal CA1, where spontaneous seizure-like activity was produced by blocking potassium ion channels and decreasing magnesium [1]. One of the main findings is that the excitatory neurons exhibit runaway excitation as the inhibitory neurons enter long-lasting depolarization block.

In this study, we employ a modified Wilson-Cowan (WC) model [2] to examine the dynamics of an EI network under similar pathological conditions. To model depolarization block, we modified the inhibitory population's activation function so that large input silences the population. Phase plane analysis shows that the network can be in as many as three different states, rest state, active state, and seizure state, where the inhibitory population enters depolarization block in the seizure state. If both populations receive a similar amount of external input, the Wilson-Cowan model predicts that the network is bistable. In this case, the network can be in either the rest state or the seizure state, where the transition to the seizure state occurs as the inhibitory population enters depolarization block. Alternatively, if the external input to the inhibitory population is small, the network can become tristable, where all three state coexist for the same parameters. The network now has to pass the intermediate state (i.e. active state) before it enters the seizure state. Such network transitions can be either oscillatory or monotonic.

We test the predictions of Wilson-Cowan model with a network of Morris-Lecar (ML) neurons. We find that the bistable state is present in the ML network, where the inhibitory neuron's firing rate peaks just before entering depolarization block (Figure 1A). This type of network dynamics has been reported in [1]. Tristability can also be observed in a ML network when the external input to the inhibitory population is reduced. The transition dynamics of the active state can be oscillatory (Figures 1B and 1C) or monotonic (data not shown) depending on the synaptic time constants as predicted by the Wilson-Cowan model. Hysteresis is observed at the transitions between different states (Figures 1A and 1B), providing evidence that the emergence of three states is a network phenomenon.

**Figure 1:**
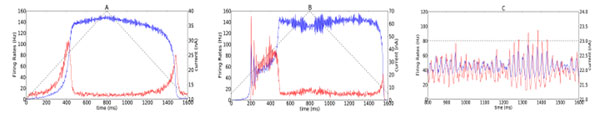
(A,B) Firing rates of excitatory (blue) and inhibitory (red) neurons. Current injection to the excitatory neurons is increased and decreased slowly as shown by the dotted lines. The network is bistable in (A) and tristable in (B). Note that the network can be in different states for the same current input, indicating hysteresis. (C) Same network parameters as in (B) but current injection to the excitatory neurons is constant to demonstrate that the network activity can be oscillatory during the active state.

## References

[B1] Ziburkus J Cressman JR Barreto E Schiff SJ Interneuron and pyramidal cell interplay during in vitro seizure-like events J Neurophy 2006 15 3948 3954 10.1152/jn.01378.2005 16554499 PMC1469233

[B2] Wilson H Cowan J Excitatory and inhibitory interactions in localized populations of model neurons Biophy J 1972 15 1 22 4332108 10.1016/S0006-3495(72)86068-5 PMC1484078

